# A Digital Platform Designed for Youth Mental Health Services to Deliver Personalized and Measurement-Based Care

**DOI:** 10.3389/fpsyt.2019.00595

**Published:** 2019-08-23

**Authors:** Frank Iorfino, Shane P. Cross, Tracey Davenport, Joanne S. Carpenter, Elizabeth Scott, Sagit Shiran, Ian B. Hickie

**Affiliations:** ^1^Brain and Mind Centre, University of Sydney, Sydney, NSW, Australia; ^2^Research and development, Innowell, Pty Ltd., Sydney, NSW, Australia; ^3^School of Medicine, University of Notre Dame, Sydney, NSW, Australia

**Keywords:** youth, transdiagnostic, mental health care, technology, ehealth, mental disorders, routine outcome monitoring

## Abstract

Mental disorders that commonly emerge during adolescence and young adulthood are associated with substantial immediate burden and risks, as well as potentially imparting lifetime morbidity and premature mortality. While the development of health services that are youth focused and prioritize early intervention has been a critical step forward, an ongoing challenge is the heterogeneous nature of symptom profiles and illness trajectories. Consequently, it is often difficult to provide quality mental health care, at scale, that addresses the broad range of health, social, and functional needs of young people. Here, we describe a new digital platform designed to deliver personalized and measurement-based care. It provides health services and clinicians with the tools to directly address the multidimensional needs of young people. The term “personalized” describes the notion that the assessment of, and the sequence of interventions for, mental disorders are tailored to the young person—and their changing needs over time, while “measurement-based” describes the use of systematic and continuing assessment of a young person’s outcomes over the entire course of clinical care. Together, these concepts support a framework for care that transcends a narrow focus on symptom reduction or risk reduction. Instead, it prioritizes a broader focus on enhancing social, health, and physical outcomes for young people and a commitment to tracking these outcomes throughout this key developmental period. Now, with twenty-first century technologies, it is possible to provide health services with the tools needed to deliver quality mental health care.

## Introduction

Mental disorders present one of the most serious public health challenges in the twenty-first century. Young people (i.e., adolescents and young adults) are particularly vulnerable with over 75% of adult mental disorders emerging before the age of 25 years (1, 2) and over 45% of the total burden of disease for those aged 10–24 years being attributed to mental ill-health (3). The high prevalence of mental disorders during adolescence and young adulthood poses a risk for future health and well-being outcomes precisely due to the timing at which they emerge (4–8). These are the chronic illnesses of young people, and if not adequately addressed, their impact can have effects that endure a lifetime (9, 10).

Consequently, we have seen major revolutions in youth mental health care, which is now set to collide with the technology boom sweeping the health sector where we have already seen a major increase in the number of mobile applications, internet-based resources, and platforms that target mental health problems (11, 12). Many of these promise to transform the way mental health care is delivered and have the potential to overcome many of the traditional barriers to conventional clinic-based care (13, 14). Here, we describe one of these solutions, a digital platform which has been codesigned with lived experience, health professionals, and services to facilitate the delivery of quality mental health care by utilizing two clinical innovations: “personalized” and “measurement-based” care.

## Personalized and Measurement-Based Care

The past decade has seen a major shift towards early intervention services and preemptive psychiatry. This shift has largely been driven by the recognition that delayed access to care and longer periods of untreated illness contribute to the complexity of treatment, development of chronic mental health problems, and secondary risks, such as function impairment and comorbid alcohol and/or other substance misuse (1, 15–20). Research to date demonstrates that young people presenting to early intervention services typically exhibit a clear need for clinical care, either due to psychological symptoms, functional impairment, or suicidal thoughts and/or behaviors, even when they do not meet traditional criteria for a mental disorder (21–26). This means that effective interventions during this time may prevent or delay the development of mental health disorders and poorer outcomes since trajectories of mental disorders and impairment are often not fixed, but instead malleable to change (27–31).

This challenge has largely driven the development of new personalized approaches (see **Box 1**) for identifying and treating common mental disorders. These approaches aim to be consistent with developmental epidemiology and neurobiology and be useful when applied in everyday clinical practice (32–35). The World Health Organizations’ mental health plan (2017–2020) emphasizes the need for mental health care to transcend the prevailing narrow medical model to address the social determinants of mental health, educational and employment opportunities, and psychosocial disability so that people can achieve their potential for health and participate fully in society. This approach reiterates that, for truly personalized mental health care in young people, a move away from categorically defined disorders toward a focus on clinically meaningful differentiations that improve outcomes are urgently needed (32, 36–38). The importance placed on diverse but related outcomes aligns with the substantial burden associated with these disorders and the needs reported by young people and their families (39, 40).

Box 1 **The nomenclature of “personalized.”**

Current uses of the term “personalized” vary depending on the medical discipline and context. A primarily biological and genetic perspective limits its use to describe unique interventions, which have been customized to an individual (e.g., personalized vaccines) (41). In psychiatry, the term personalized has been replaced with terms such as “stratified,” to subtype illnesses on the basis of salient treatment-relevant characteristics (36) or “precision” medicine to place greater emphasis on the exactness of measurement (42). A broader, yet related, concept is “person-centered” medicine, which is commonly used to describe a holistic view of the individual with an emphasis on the role of the person in treatment (43). The term “personalized” in this paper aims to encompass various facets from the definitions described above to describe broadly the notion that the assessment of, and the sequence of interventions for, mental disorders are tailored to the individual, and their changing needs over time.

Similarly, measurement-based care as a health service quality improvement strategy may be particularly suited to mental health care. Measurement-based care involves the systematic and continued assessment of an individual’s outcomes over the entire course of clinical care. It supports better-informed and highly personalized clinical decisions about treatment throughout the entire episode of illness (44, 45). Many reviews have demonstrated the effectiveness of these approaches including faster symptom improvement and lower likelihood of deterioration during care (46–50). Namely, monitoring an individual’s progress during care can reduce deterioration and improve treatment effects by notifying individuals and clinicians of positive and negative changes following a particular treatment (51, 52). This facilitates the opportunity to alter the treatment plan accordingly and actively engage young people (and their families) who may have disengaged or are not adhering to treatment.

When combined with the concept of personalized mental health care, the use of measurement-based care has the potential to improve outcomes in real-world settings (45). This framework differs from manual-based approaches and interventions by moving away from clinical decisions based on the “average” patient to a focus on the individual by routinely monitoring their outcomes. The measurement-based feedback helps to detect unmet care needs and enables the earlier identification of other markers of need. This begins with a broad assessment to get a complete and personalized overview of the young person’s health and well-being, avoiding a narrow focus on symptom reduction or risk identification. Secondly, these measurements are repeated overtime across these broad domains to determine specific personalized changes over the course of treatment. Identifying these personalized changes in response to an intervention can help determine whether an intervention should be adapted or whether a change in the outcome focus is needed.

## A Digital Solution—The Innowell Platform

The Innowell Platform is a configurable digital tool that aims to facilitate personalized and measurement-based care within a mental health service. It is one of the first platforms developed that puts into practice many of the innovative and emerging models of mental health care discussed internationally (34, 38, 40, 53). While here we focus specifically on young people, the Platform has been designed for, and is used in, a range of other service populations (e.g., children and families, veteran community, and older adults), and many of the concepts discussed below also generalize to these other populations. Importantly, the Platforms design has been informed through an ongoing process of participatory design with lived experience, health professionals, and service staff (including administration and management) across different service populations (e.g., headspace services, Opens Arms veterans and families counseling service) (54), and is the current focus of a clinical trial across these different service settings (55).

The Platform assists with the assessment, feedback, management, and monitoring of their mental ill health and maintenance of well-being by collecting personal and health information from a young person, their clinician(s), and supportive others. This information is stored, scored, and reported back to the young person, their clinicians, and the service provider to promote genuine collaborative care (56, 57). The clinical content is determined by the health service who invites the young person to use the Platform. The Innowell Platform does not provide stand-alone medical or health advice, risk assessment, clinical diagnosis, or treatment. Instead, it guides and supports (but does not direct) young people and their clinicians to decide what may be suitable care options. Importantly, all care aligns with the existing clinical governance (e.g., policies and procedures) of the service provider.

The Platform facilitates personalized and measurement-based care within a mental health service by enhancing key processes, which themselves may not be new, but their combined use and integration with face-to-face services is. Specifically;

### Assessment

The assessment uses a multidimensional outcomes framework to cover domains of social and occupational function, self-harm and suicidal thoughts and/or behaviors, physical health and concurrent alcohol and/or other substance misuse, as well as illness type, stage, and trajectory (e.g., symptoms, diagnoses, treatment history) (58). The Platform can present a set of assessments; however, the exact content or makeup of these assessments are configured by the service using the Platform so that the content can be modified to address the needs of any population or setting. The Platform currently contains a library of evidence-based questionnaires for services to choose from, which are commonly used in youth mental health studies and clinical practice (e.g., Overall Anxiety Severity and Impairment Scale, and Kessler Psychological Distress Scale); however, this library will continue to expand as additional questionnaires are added. Also, developmental considerations have been made for younger populations, whereby it is possible to use age appropriate stimuli (e.g., pictures) for assessments.

To achieve a greater level of personalization, these assessments can also be tailored to individuals by demographic (e.g., age, gender) or clinical information (e.g., endorsement of self-harm, depression score). The Platform also facilitates the integration of information collected for other sources; namely, health professionals who might be seeing the young person in face-to-face care, a supportive other(s) (e.g., parent or guardian) who the young person can invite to fill out information about them, or devices (e.g., activity monitors such as Fitbit) to provide a more detailed understanding about underlying pathophysiology and multidimensional outcomes.

### Feedback

**Figure 1:**
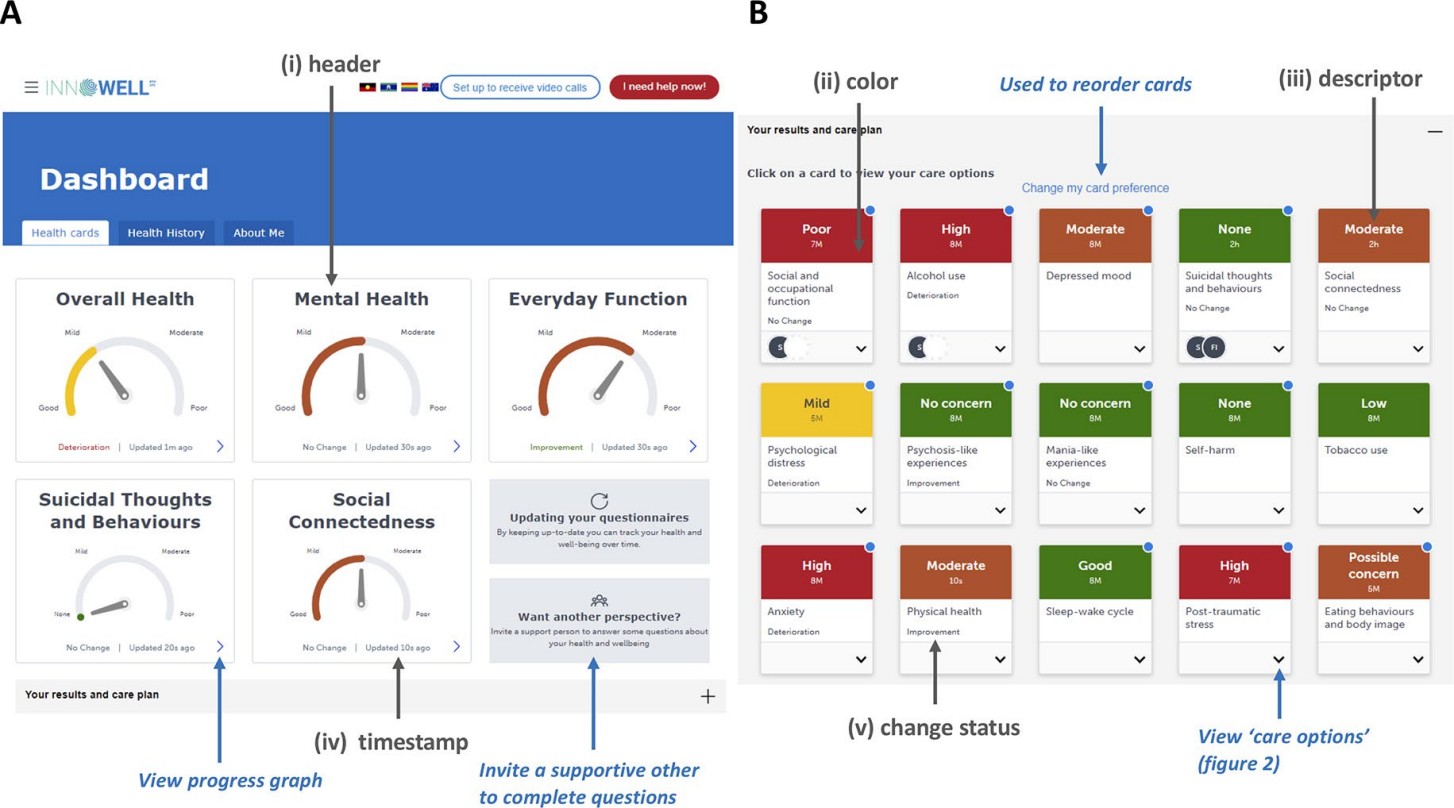
The dashboard of results for the Innowell Platform. Panel **(A)** shows the summary dashboard which provides a broad overview of the young person’s results for key health domains. Panel **(B)** shows the more detailed dashboard (i.e., “your results and care plan” section expanded), whereby all of the results from the broad assessment are displayed on different “health cards.” Each domain is accompanied by a scale, the current result (represented using color and a dial), and an indication of personalized change. The Platform allows for the customization of specific domains, questions, and algorithms for this section. The use of color here is to make it easier for young people and clinicians to figure out what might be tracking well or not so well. The color is accompanied by a text description (e.g., high) as well as a timing (e.g., 3M, equal to “3 months”), which is used to indicate how recent the current result is. The middle of the health card presents the title and includes the personalized change status in text (e.g., improvement). The circles presented on the “suicidal thoughts and behaviors” health card provides an example of how the Platform communicates whether or not an intervention is currently active for a particular domain. Here, the initials of the treating clinician are displayed along with the young persons initials to indicate they are working on this together. Gray text and arrows highlight key components described the feedback section of this paper; blue text and arrows highlight other key components. Please note that the image presented here displays the Platform as it exists at the date of this publication, and is subject to further development and refinement.

The dashboard is used to feedback results to the young person, support persons (e.g., family members), clinical services, and clinicians (**Figure 1**). The Platform automatically processes assessment results using a set of algorithms that score and interpret the responses and data. These results are presented on a single page using a set of “cards,” which are presented using either gauges (panel **A**) or text (panel **B**). Each card (gauge or text) contains the same type of information (see **Figure 1**); i) “a header” indicating the domain (e.g., depressed mood); ii) “color,” which is used to communicate whether a result is good (green) or poor (red); iii) “a descriptor,” which provides feedback about the result (e.g., anxiety is high); iv) “a time stamp,” which indicates when a domain was last updated (e.g. 5M = 5 months); and v) “a change status” to compare the current result to the previous result (e.g., “no change” or “improvement”). Each “card” aims to summarize a young person’s current outcome within a domain, and together, these cards provide an overall view of the totality of needs across each domain within the multidimensional outcomes framework. The use of color and presenting these cards altogether facilitates the ease and efficiency of quickly understanding where the areas of high or low concern might be. These cards can be reordered by the young person to reflect their preferences regarding which domains are particularly important to them versus those that are less important (see **Figure 1**, panel **B**). This is an important feature that indicates how the Platform can be used to facilitate communication between the young person and health professional for the management of their mental ill-health and well-being.

### Management

**Figure 2:**
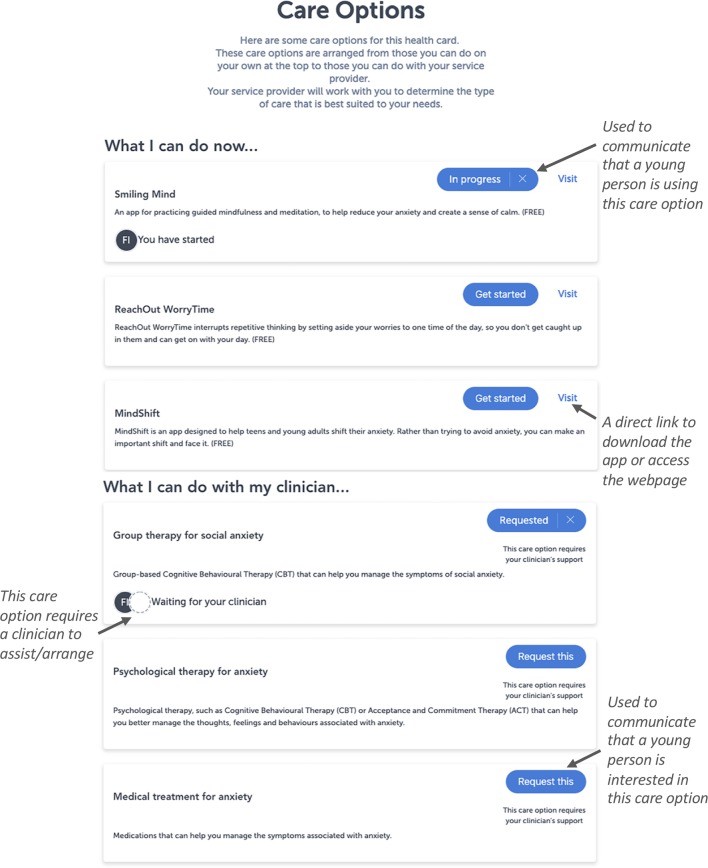
The more detailed view of the dashboard of results, which facilitates shared-care planning and the management of mental ill-health. The figure presents the care options that are available for this particular domain and shows how the blue buttons can be used to facilitate shared decision-making. These care options are customized according the health service using the Platform, so that it accurately reflects their clinical offering. Each care option is accompanied by a title, a description, an action button, and a status icon on the bottom left. Specific actions can be performed for these care options using the buttons on the right of each care option. In this example, the circle with an “FI” on the “smiling mind” care option is used to indicate that this young person has started to use the app to address their anxiety. The circle with an “FI” and dotted circle on the “group therapy for social anxiety” care option is used to indicate that the young person is interested in this intervention and would like to speak to their clinician about it. Please note that the image presented here displays the Platform as it exists at the date of this publication, and is subject to further development and refinement.

The Platform also provides the capacity for shared decision-making between the young person and their support person(s), and health professionals to facilitate the management of mental ill-health. The Platform presents specific care options that are available for the entire range of domains assessed by the assessments and presented on each card on the dashboard. These care options are divided into two categories; “what I can do now,” which presents apps or etools that are accessible immediately to a young people; and “what I can do with my clinician,” which presents the range of clinical interventions provided by a service that require a clinician or services support to do (**Figure 2**). Each care option is accompanied by a set of actions that facilitate communication between the young person and their clinician(s). For example, these actions may be used by the young person to indicate they are interested in a particular care option offered by the service [e.g., cognitive behavior therapy (CBT) for depression]. This increases the transparency about who is working with the young person, what the target for intervention is, and how the young person and clinician(s) will work together to address it.

Specific triggers can be set up based on the responses provided during the assessment to help a service manage specific concerns. For example, if a young person reports current suicidal thoughts and/or behaviors, the Platform will present an immediate pop-up that notifies the young person that they can get immediate help from specific helplines or services, and a notification will also be sent to a health professional at the service so that they can respond in an appropriate way (e.g., telephone follow-up, safety planning, schedule a sooner face-to-face appointment). This notification will remain active (e.g., flagged in the Platform) until a response is made by the service. The types of triggers, their thresholds, and service responses to manage them can be configured to meet the specific needs of a service population or setting.

### Monitoring

The multidimensional assessment is repeated over time to track changes across multiple domains, and the results for each domain over time are presented in two ways to communicate change. The first is through the use of text (i.e., “improvement,” “no change,” “deterioration”) and color (green text for positive results, red text for negative results) on each card of the dashboard (see **Figure 1**), and the second is through a graphical display, which plots the outcomes for each domain over time. The assessments can be repeated at regular intervals (e.g., every month), whereas there are summary questions (see **Figure 1**, panel A) that can be completed at more regular intervals (e.g., daily) to provide a more fine-grained summary of changes in mental health and functioning.

## Applied Use Within a Mental Health Service

**Figure 3:**
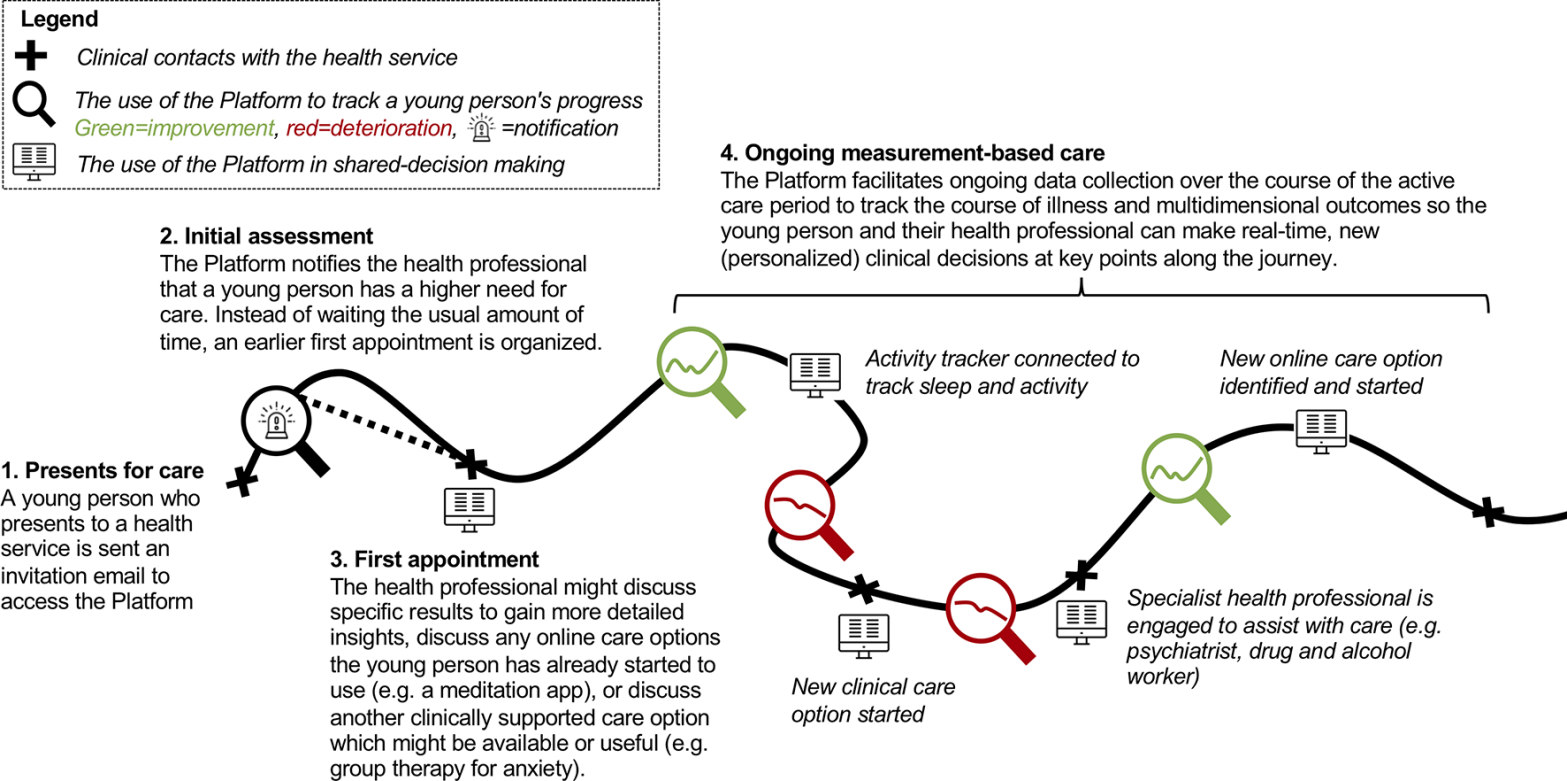
Illustrative example of a young person’s journey through care. The figure shows how the Platform can be used at different points along the care journey to facilitate measurement-based care to drive personalized treatment decisions. Specifically, in this example, the Platform is being used at entry to care to identify young people reporting higher levels of suicidality, who may need to “skip” the waitlist. The first appointment (much like proceeding clinical contacts) can be used to engage in usual face-to-face clinical care or enhanced by the Platform enhanced by the Platform (as described in point 3 above). As care progresses, ongoing data collection and tracking are used to make personalized treatment decisions, such as connecting to an activity tracker to better understand a young person's sleep wake cycle in relation to their mood, start a new clinical intervention or engage a specialist due to a deterioration in outcomes, or engage in an online intervention when outcomes start to improve. “Computer” by Nikita Kozin from the Noun Project; “Alarm” icon made by Freepik from www.flaticon.com.

The Innowell Platform enables the delivery of enhanced care that builds on the usual processes provided by the services by facilitating systematic assessment and the promotion of clinical care within multidisciplinary team environments. The application and use of the Innowell Platform within a mental health service will vary depending on the setting and population. Each service has distinct pathways into and out of care and will vary in terms of their service offering. This means that how the Platform is specifically used by a service is determined on a case-by-case basis through an ongoing process of implementation, which involves understanding and mapping the care pathways for a mental health service and asking the critical question: how can each of the components (i.e., assessment, feedback, management, and monitoring) be used to enhance those pathways? For illustration purposes, a commonly used model for how the Platform is used by young people and health professionals is provided in **Figure 3**. The figure shows how technology can be used to enable typical care pathways and processes by improving the personalization and use of measurements to guide clinical decision-making.

Since young people are more likely to present with multidimensional needs (59), health service strategies should be in place to identify and respond to a range of health, educational, employment, and social issues (60). Here, services can make use of the Platforms assessments at initial presentation and over time to identify known predictors of outcomes or subgroups of individuals who have differential care needs. A higher severity of needs tends to be associated with poorer outcomes over time, and so strategies that address these problems may be able to improve long-term outcomes (61). A recent systematic review demonstrated that of the few studies that assessed multiple health domains (e.g., mental health, alcohol use, sleep) in primary care, screening facilitated the opportunity to provide targeted interventions and led to better health outcomes (62, 63).

Previous prototypic work demonstrates the utility of the Platform, integrated with a youth mental health service, to enable an appropriate and timely response for young people reporting higher levels of suicidality (64). The use of the Platform meant that clinicians could utilize the clinical details from the multidimensional assessment, such as the presence of comorbid issues, and a history of suicide plans and attempts to make informed clinical decisions. Further prototypic work also demonstrated how it can also be used to more broadly assess the totality of a young person’s needs before a one-on-one assessment (face-to-face or video visit) and enable clinicians to move away from traditional evaluations towards more detailed data-driven assessments (65).

The Platform facilitates the development and evaluation of specific, integrated care packages across multiple domains, which may be needed to reduce the morbidity and mortality due to mental disorders (66, 67). Inherent in these approaches is moving beyond symptom and risk management to an emphasis on improving outcomes and reduce secondary risks. These approaches are particularly suitable for young people with many co-occurring conditions since interventions can be effectively coordinated in a way that addresses their complex needs (68). This also facilitates the use of integrated or conceptual unified approaches that incorporate multiple sources of information about causal or maintaining factors to develop interventions that target these processes (69, 70). Some patients do deteriorate during treatment, and some require long-term care, so being able to identify an individual’s progress during treatment may facilitate decisions about the effectiveness of current interventions and the timing of new ones (71). We have previously shown that young people with complex needs often leave care too early, before they have improved (72, 73). As services do not routinely track clinical or functional outcomes, individuals may end up being overtreated, undertreated, or not treated at all. Practically, this can result in a worsening of the underlying syndrome (74), acute presentations to emergency departments, overutilization of crisis services, greater physical health comorbidity, ongoing functional impairment (73), as well as alcohol and/or other substance misuse (75).

## Conclusion

The youth mental health landscape has undergone dramatic transitions over the past decade, particularly in terms of awareness, access, and resource allocation. These advances are a crucial step in the right direction for addressing mental disorders and their associated burden in young people. The next important step in this revolution is to ensure that young people who access services receive quality, personalized, and measurement-based mental health care that addresses their multitude of needs early in life so that they can lead fulfilling and contributing lives later in adulthood. With major mental health reform on the global agenda, here, we present a digital platform that provides health services with the essential tools needed to deliver quality mental health care.

## Author Contributions

All authors discussed the evidence and contributed to the writing of this manuscript. FI drafted the manuscript. All authors contributed to and have approved the final manuscript.

## Funding

This work was partially supported by grants from the National Health & Medical Research Council (NHMRC) including Centre of Research Excellence (No. 1061043), and Australia Fellowship (No. 511921 awarded to IH). This work is also supported by Project Synergy. In June 2017, the Australian Government Department of Health and InnoWell Pty Ltd entered into a 3-year funding agreement including a series of collaborative research trials known as Project Synergy that are continuing the codesign, development, implementation, and evaluation of the InnoWell Platform.

## Conflict of Interest Statement

ES is the medical director of Young Adult Mental Health Unit, St Vincent’s Hospital Darlinghurst; discipline leader of Adult Mental Health, School of Medicine, University of Notre Dame; research affiliate at The University of Sydney; and a consultant psychiatrist. She has received honoraria for educational seminars related to the clinical management of depressive disorders supported by Servier and Eli-Lilly pharmaceuticals. She has participated in a national advisory board for the antidepressant compound Pristiq, manufactured by Pfizer. She was the National Coordinator of an antidepressant trial sponsored by Servier. IH was a commissioner in Australia’s National Mental Health Commission from 2012 to 2018. He is a co-director of the Health and Policy at the Brain and Mind Centre (BMC) University of Sydney. The BMC operates an early-intervention youth services at Camperdown under contract to headspace. IH has previously led community-based and pharmaceutical industry-supported (Wyeth, Eli Lily, Servier, Pfizer, AstraZeneca) projects focused on the identification and better management of anxiety and depression. He is a board member of Psychosis Australia Trust and a member of Veterans Mental Health Clinical Reference group. He is the chief scientific advisor to, and an equity shareholder in, InnoWell. InnoWell has been formed by the University of Sydney and PwC to administer the $30M Australian Government Funded Project Synergy. Project Synergy is a 3-year program for the transformation of mental health services through the use of innovative technologies.

The remaining authors declare that the research was conducted in the absence of any commercial or financial relationships that could be construed as a potential conflict of interest.
